# Analytical profiling of mutations in quinolone resistance determining region of *gyrA* gene among UPEC

**DOI:** 10.1371/journal.pone.0190729

**Published:** 2018-01-04

**Authors:** Lesley R. Varughese, Meenakshi Rajpoot, Soniya Goyal, Ravinder Mehra, Vinod Chhokar, Vikas Beniwal

**Affiliations:** 1 Department of Biotechnology, Maharishi Markandeshwar University, Mullana, Ambala, India; 2 Department of Bio & Nano Technology, Guru Jambheshwar University of Science & Technology, Hisar, Haryana, India; Animal and Plant Health Agency, UNITED KINGDOM

## Abstract

Mutations in *gyrA* are the primary cause of quinolone resistance encountered in gram-negative clinical isolates. The prospect of this work was to analyze the role of *gyrA* mutations in eliciting high quinolone resistance in uropathogenic *E*.*coli* (UPEC) through molecular docking studies. Quinolone susceptibility testing of 18 *E*.*coli* strains isolated from UTI patients revealed unusually high resistance level to all the quinolones used; especially norfloxacin and ciprofloxacin. The QRDR of *gyrA* was amplified and sequenced. Mutations identified in *gyrA* of *E*.*coli* included Ser83Leu, Asp87Asn and Ala93Gly/Glu. Contrasting previous reports, we found Ser83Leu substitution in sensitive strains. Strains with S83L, D87N and A93E (A15 and A26) demonstrated norfloxacin MICs ≥1024mg/L which could be proof that Asp87Asn is necessary for resistance phenotype. Resistance to levofloxacin was comparatively lower in all the isolates. Docking of 4 quinolones (ciprofloxacin, ofloxacin, levofloxacin and norfloxacin) to normal and mutated *E*.*coli* gyrase A protein demonstrated lower binding energies for the latter, with significant displacement of norfloxacin in the mutated GyrA complex and least displacement in case of levofloxacin.

## Introduction

Quinolones comprise broad-spectrum antibiotics successfully used over the years for the treatment of many infections. The greater potency and versatility of these drugs paved the way for effective therapy of many diseases like urinary tract infections, osteomyelitis, pneumonia and gastrointestinal diseases. However, their extensive use and misuse has also led to the emergence and spread of resistance among bacteria [1, 2].

DNA gyrase and topoisomerase IV (type II topoisomerases) are the prime targets of quinolones. DNA gyrase, a tetramer of two A and two B subunits, encoded by the *gyrA* and *gyrB* genes respectively, is responsible for uncoiling the intertwined circles of double-stranded bacterial DNA that arise after each round of replication, hence relieving topological stress. Quinolones obstruct the normal process of DNA synthesis by establishing a stable ternary complex with gyrase and DNA [3, 4]. The quinolone-gyrase-DNA complex prevents the broken strands of DNA from resealing thus leading to accumulation of double stranded breaks, disruption of cell growth and finally apoptosis [5]. DNA gyrase is the main target in gram-negative organisms (for example, *Escherichia coli*) while quinolones target topoisomerase IV in gram-positive organisms (for example, *Streptococcus pneumoniae*) [6].

Intensive research has recognized mutations in chromosomal genes that lead to alterations in the drug targets (DNA gyrase and topoisomerase IV) to be the major cause of quinolone resistance encountered in clinical isolates [7]. Mutations occur frequently on the chromosomal genes encoding gyrase and topoisomerase IV. They mostly include *gyrA* that code for the A subunit of gyrase (GyrA) in gram-negative bacteria and *parC* that code for ParC subunit of topoisomerase IV in gram-positive bacteria. Studies have revealed that these mutations predominate in conserved regions or “hot spots” known as the quinolone-resistance-determining region or QRDR, involved in DNA binding. In GyrA, the QRDR comprise amino acids between 67 and 106. The QRDR is located close to tyrosine 122 that binds covalently to DNA during strand breakage and rejoining [8]. The most common mutations discovered in gram-negative bacteria like *E*.*coli*, *Shigella*, *Citrobacter* and *Pseudomonas* were at codon 83 and 87 in GyrA [2, 9].

Since their release in 1980’s, quinolones like ciprofloxacin, ofloxacin, levofloxacin, gatifloxacin etc have been used to treat urinary tract infections due to their gram-negative activity [10]. Incidentally, this has led to the spread of resistance in urinary pathogens like *Escherichia coli*, *Klebsiella* and *Enterobacter* [11]. Studies from different parts of India on UTI cases have reported high quinolone resistance trend among gram-negative enteric pathogens [12, 13].

Many studies have reported mutations in QRDR of *gyrA*, *gyrB*, *parC* and *parE* to be solely responsible for quinolone resistance. It is imperative to figure out whether these mutations are responsible for affecting the drug affinity and susceptibility profile of common pathogens. In the present venture, efforts were made to analyze gyrase mutations in *E*.*coli* that could be responsible for increased quinolone resistive mechanisms among enteric pathogens. Docking studies revealed displacement of quinolone binding site in mutated protein complex which resulted in lower binding energy as compared to the normal one. This could be the reason for the high resistance pattern evident in this study.

## Materials and methods

### Strains, media and antibiotics

The isolation and biochemical identification of eighteen *E*.*coli* strains selected in this study has been previously described in [12]. All the media used were purchased from HiMedia Laboratories Pvt. Ltd. Ciprofloxacin, levofloxacin, norfloxacin and ofloxacin (Cipla Ltd, Mumbai) were the quinolones used in this study.

### Antimicrobial susceptibility testing

Antimicrobial susceptibility testing was performed on Muller Hinton Agar by agar dilution method to determine the minimum inhibitory concentration (MIC) at varying concentrations of quinolone antibiotics as per guidelines [14]. The MICs for each isolate were determined as per the interpretive standards defined by Clinical and Laboratory Standards Institute M100-S23 [15].

### Isolation of genomic DNA and 16S rDNA sequencing

Isolation of DNA was accomplished using HiPurA™ bacterial genomic DNA purification kit following manufacturer’s instructions. The eluate was then stored at -20°C. Amplification of 16S rDNA was done by PCR using universal primers (Spectrum Technologies) -8F (5’-AGAGTTTGATYMTGGCTCAG-3’) and 1495R (5’-CTACGGCTACCTTGTTACG-3). PCR reaction mixture for 25μl was as follows: 12.5μl Master Mix (1X) (#K0171-Thermo Scientific), 4 μl forward primer (20 pmole), 4 μl reverse Primer (20 pmole), 2 μl DNA, 2.5μl nuclease free water. The components of the reaction mixture were placed in a thermal cycler (Applied Biosystems) that was programmed for 35 cycles in the following conditions: Initial denaturation (94°C)-5 minutes, denaturation (94°C)-1 minute, annealing (54°C)-1 minute, extension (72°C)-1 minute and final extension (72°C)-7 minutes. The PCR amplicons were sequenced by ABI Prism 3730 *xl* DNA analyzer (Applied Biosystems) using Sanger’s (dideoxynucleotide chain termination) method. The 16SrDNA sequences were checked for sequence similarity using BLAST and were submitted to GenBank for accession numbers.

### Amplification and sequencing of *gyrA* QRDR

PCR amplification was done using primers obtained from Integrated DNA technologies- gyrA11753 (5’-GTATAACGCATTGCCGC-3’) and gyrA12004 (5’- TGCCAGATGTCCGAGAT-3). The reaction mixture for 25 μl was as follows: 12.5μl Master Mix (1X), 2μl forward primer (20 pmole), 2 μl reverse primer (20 pmole), 2 μl DNA, 6.5μl nuclease free water. The components of the reaction mixture were processed in a thermal cycler (Applied Biosystems) for 35 cycles in the following conditions: Initial denaturation (94°C)-7 minutes, denaturation (94°C)-1 minute, annealing (46°C)-1 minute, extension (72°C)-30 seconds and final extension (72°C)-10 minutes. The PCR amplicons were sequenced in one direction by ABI Prism 3730 *xl* DNA analyzer (Applied Biosystems) using Sanger’s (dideoxynucleotide chain termination) method.

#### Genetic analysis

The *gyrA* sequences were checked for similarity with reference gene MG1655 (*E*.*coli* K-12) using NCBI’s BLAST. Multiple sequence alignment (MSA) was then performed using the online software Clustal Omega. The sequences were translated into amino acid codon using Expasy translation tool. The protein sequences were then checked for similarity in BLAST and again aligned using Clustal Omega.

### Docking of quinolone using AutoDock 4.2.5

The X-ray crystal structure of *E*.*coli* gyrase A protein (pdb ID– 1AB4) was obtained from RCSB protein data bank. The ligand structures- ciprofloxacin, norfloxacin, ofloxacin and levofloxacin with identification numbers 2764, 4539, 4583 and 149096 respectively; were available from NCBI pubchem (www.pubchem.ncbi.nlm.nih.gov). Mutations at positions 83 (Ser→Leu) and 87 (Asp→Asn) of amino acid sequence were incorporated into the normal protein. Docking was performed with the ligands using Autodock4.2.5 for normal and mutated strains. Binding energy was calculated in Autodock for both the complexes.

## Results and discussion

The MICs and GenBank accession numbers of the *E*.*coli* strains have been depicted in Table 1. The choice of *E*.*coli* in this study is attributed to its predominance as an uropathogen [12]. Majority of the UPEC isolates (89%) exhibited resistance to all the quinolones tested. Resistance phenotype was quite high (MIC>1024mg/L) for norfloxacinin (44%) among the isolates. Extensive prescriptions of second generation quinolones like norfloxacin, ciprofloxacin and ofloxacin for complicated and uncomplicated UTIs; along with inapt use, under dosing and unawareness could be reasons for this trend [16]. However, levofloxacin and ofloxacin (16-128mg/L) showed comparatively lower level of resistance. Enhanced gram positive activity of levofloxacin and medication of ofloxacin for other urogenital cases may be reasons [17].

**Table 1:** Accession numbers, quinolone susceptibility and GyrA mutations of *E*.*coli* isolates.

Strain	Accession number	MIC (mg/L)				GyrA mutations
		N	C	L	O	
**38**	KP276747	<2	<2	<2	<2	S83L
**55**	KP276762	<2	<2	<2	<2	S83L
**27**	KP276740	512	64	64	128	S83L
**41**	KP276750	1024	256	64	256	S83L, D87N
**21**	KP276734	16	16	16	16	S83L, A93G
**8**	KP276721	2048	256	64	128	S83L, D87N
**22**	KP276735	512	128	64	128	S83L, D87N, A93G
**44**	KP276753	512	512	128	256	S83L, D87N, A93G
**39**	KP276748	1024	256	64	128	S83L
**47**	KP276756	1024	256	64	64	S83L, D87N
**20**	KP276733	2048	256	64	128	S83L, D87N
**7**	KP276720	256	256	32	64	S83L, D87N
**14**	KP276727	512	256	128	256	S83L, D87N
**12**	KP276725	256	256	64	128	S83L, D87N
**18**	KP276731	>3500	512	64	128	S83L, D87N
**6**	KP276719	256	128	32	64	S83L, D87N, A93G
**15**	KP276728	>3500	512	128	256	S83L, D87N, A93E
**26**	KP276739	1024	256	32	128	S83L, D87N, A93E; P79H, H80Q, T88S, R91H

**Fig 1:**
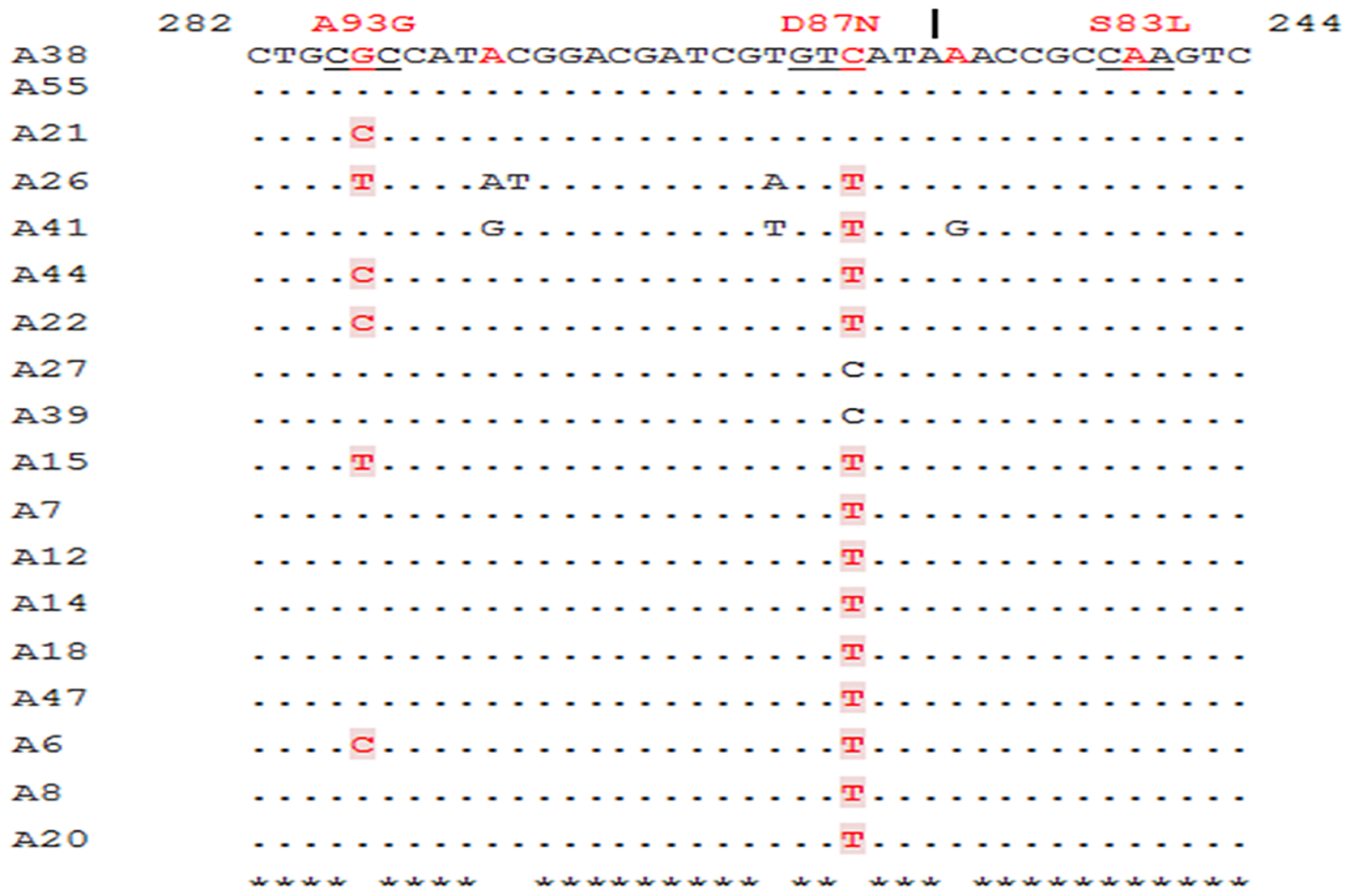
DNA sequence similarity among *gyrA* QRDR of *E*. *coli* strains. Substitutions leading to amino acid changes are highlighted. Amino acid mutations are mentioned above specific codon. A38, A55 -sensitive strains, A21- intermediate & remaining strains are resistant. Identical nucleotides are dotted while those different from sensitive strain are highlighted.

Sequencing of *gyrA* QRDR of 18 *E*.*coli* strains and its alignment with reference strain MG1655 from nucleotides 101 to 319 revealed mutations at codons 83, 87 and 93 (Table 1). All the strains, including sensitive strains (A38 and A55) possessed Ser83 to Leu substitution (G248A). Double mutations, Ser83 to Leu (G248A) and Asp87 to Asn (C259T) were seen in 8 strains. Triple mutants had an additional mutation- Ala93 to Gly/Glu (G278C/T), and this was observed in 5 strains (Fig 1).

Association between *gyrA* mutations and quinolone resistance was substantiated through dendogram analysis (S1 Fig). Strains A26 and A15 (norfloxacin MICs ≥ 1024mg/L) comprise group I and are similar with respect to the mutations they possess (S83L, D87N and A93E). Resistance pattern followed was norfloxacin> ciprofloxacin > ofloxacin > levofloxacin. They form a cluster with group II that include A22 and A44, both of which possess triple mutations (S83L, D87N and A93G) and identical resistance levels. A21 and A6 form sub-group III and cluster with A8 and A20 (sub-group IV) that have exactly same MICs. Another cluster can be presumed between A27 and A39 (high MICs) and sensitive strains A55 and A38. This may be related to the presence of S83L mutation.

The existence of Ser83 to Leu mutation in sensitive strains A38 and A55 (MIC<2mg/L) and in moderately susceptible strain A21 (MIC 16mg/L) contradicts earlier reports of its positive influence in acquiring quinolone resistance [18–20]. We also found substitution in Ser83 to be identical in all the samples. All the resistant strains had double (S83L, D87N) or triple (S83L, D87N, A93G/E) *gyrA* mutations. Norfloxacin MICs for most of the double mutants were greater than 1024mg/L and ciprofloxacin MICs 256mg/L. Double mutations in gyrase are thought to reduce the affinity of the gyrase-DNA complex for quinolones [21]. Sequencing of QRDR region by Namboodiri et al. [22] observed that isolates with an additional *gyrA* substitution Asp87→Asn were resistant to both ciprofloxacin as well as nalidixic acid. Alteration of Ala93 to Gly could be responsible for moderate susceptibility in A21 however its role in triple mutants is yet to be understood. Strains with S83L, D87N and A93E demonstrated norfloxacin MICs ≥1024mg/L. Resistance to levofloxacin was comparatively lower in all the isolates.

**Fig 2:**
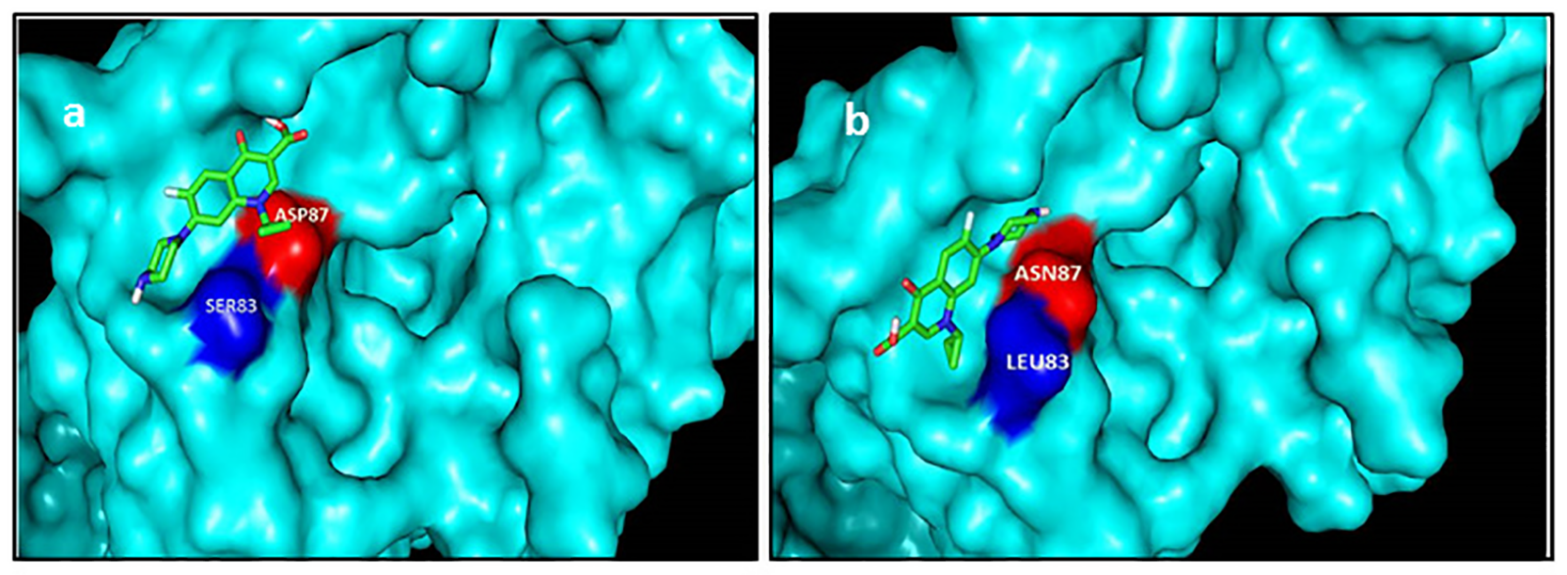
Docking structures of ciprofloxacin with (a) normal and (b) mutated GyrA QRDR. Displacement of the ligand is evident in (b) when Asp87 was changed to Asn87.

Docking results of quinolones with both normal and mutated *E*.*coli* GyrA are represented in terms of binding energies. Higher binding energy signifies stronger interaction between the ligand and protein. Ciprofloxacin-GyrA docking structure is depicted in Fig 2. Ciprofloxacin was seen to only interact with Asp87 in wild type protein (Fig 2a). Visualization of ciprofloxacin-GyrA complex showed displacement of the ligand in case of mutated GyrA (Fig 2b). The binding energy was -4.84 kcal/mol for the wild type protein complex. Hydrogen bonds involved in this case were Ala117- 2.9A°, Ser116- 2.69A° and Ala118- 3.99A°. However, the binding energy was reduced to -4.4. kcal/mol in case of mutated protein complex. This could be a consequence of the loss of one hydrogen bond (Ala 118). The drug does not bind with Asn87.

**Fig 3:**
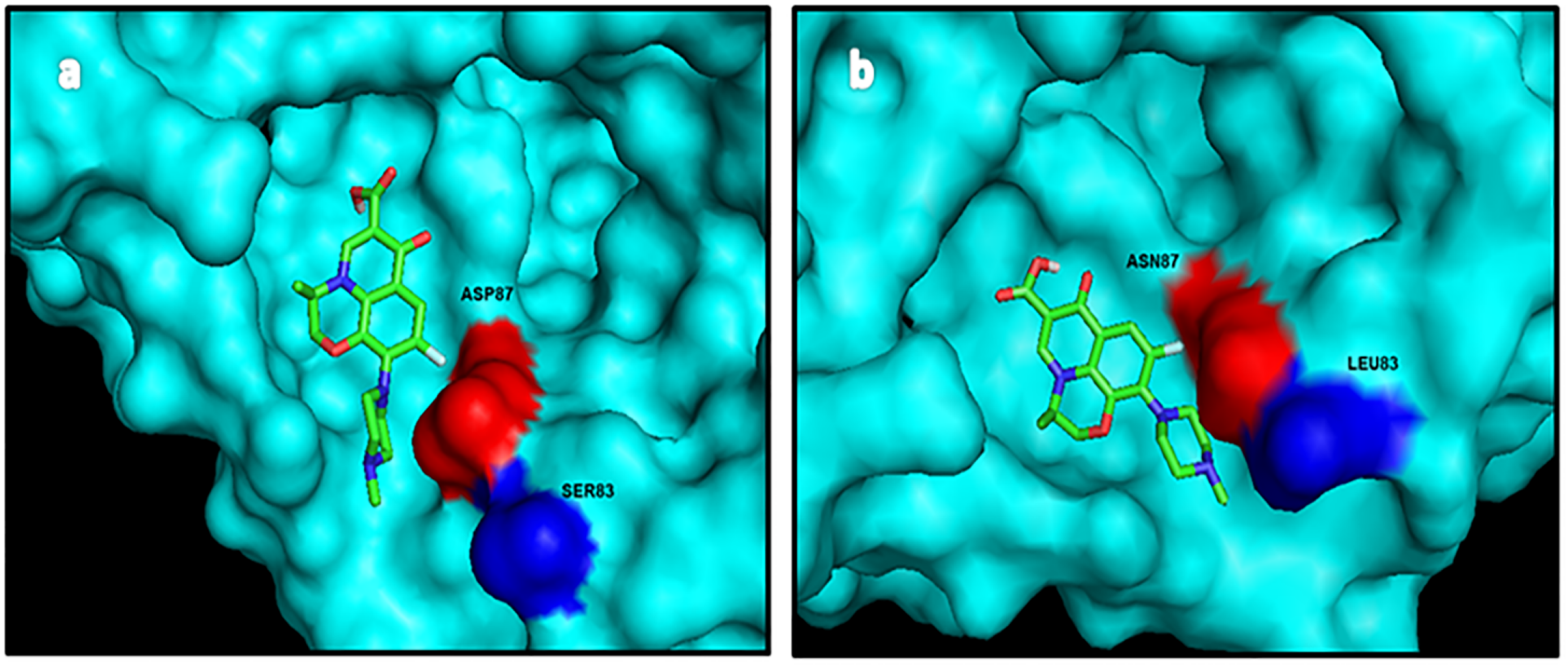
Docking structures of ofloxacin with (a) normal and (b) mutated GyrA QRDR.

**Fig 4:**
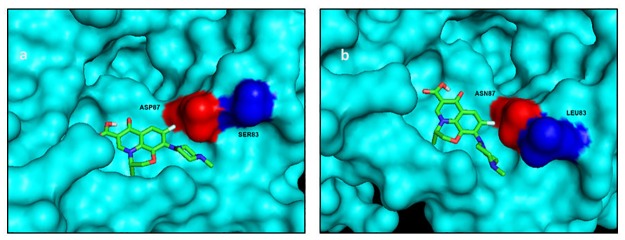
Docking structures of levofloxacin with (a) normal and (b) mutated GyrA QRDR.

**Fig 5:**
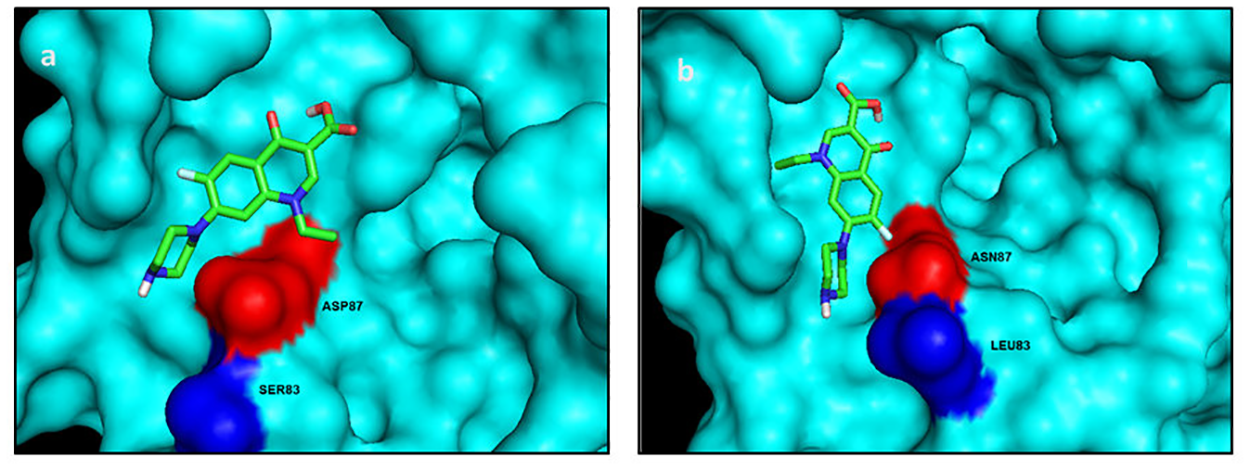
Docking structures of norfloxacin with (a) normal and (b) mutated GyrA QRDR.

Docking of ofloxacin with mutated *E*.*coli* GyrA resulted in a binding energy of -6.92 kcal/mol, which was lower than the binding energy of the wild type complex (-7.31 kcal/mol). The ligand in case of mutated GyrA was also observed to be shifted (Fig 3). Hydrogen bonds involved were Asp87, Arg91 and Gln94. Although the binding energy was found to be lower for the levofloxacin-mutated GyrA complex (-7.18kcal/mol) with respect to the normal protein complex (-7.32 kcal/mol), docking studies have not displayed much displacement (Fig 4). Asp87, Ser97, Arg91 are hydrogen bonds involved in this complex. Significant displacement of norfloxacin in the mutated GyrA docking structure could be attributed to lower binding energy (-5.73kcal/mol) when compared to a binding energy of -5.93kcal/mol in the wild type complex (Fig 5). Hydrogen bonds include Asp87, Arg91 and Ala117.

Docking studies have provided a clue for the remarkably high quinolone resistance phenotype seen in our strains. The lack of any interaction between quinolones and Ser83 of GyrA could be reason for low MIC in strains with Ser83Leu mutation. It is only in the presence of a second mutation (Asp87Asn) that strains develop high resistance. However, the absence of any strain with D87N alone confirms that mutation in Ser83 is indeed important for step-wise resistance acquisition. Additionally, studies have reported resistance in strains with other Ser83 mutations like Phe/Tyr [23, 24]. Shifting of the binding site in mutated GyrA could lower the affinity of the protein for the drug as evidenced by loss of hydrogen bond, thus lower susceptibility towards ciprofloxacin. Vashist et al. [23] analyzed docking of ciprofloxacin with mutated GyrA of *E*.*coli* and found weaker interaction between them as compared to the normal protein. The comparatively higher MIC values of ciprofloxacin and norfloxacin with respect to ofloxacin and levofloxacin could be explained by its significant displacement from the QRDR of GyrA.

## Conclusion

It is prudent to be acquainted with the susceptibility profile of bacteria in a geographical area which not only helps in prescription of the right drug for any infection but also encourages the judicial use of antibiotics. This prompted us to isolate enteric pathogens from UTI patients and study their resistance pattern and the molecular mechanism that is responsible for it. We found *E*.*coli* to be the leading uropathogen in our samples which is trend in other parts of India also [25, 26].

Testing of *E*.*coli* isolates for susceptibility to quinolones revealed remarkably high resistance levels, especially toward norfloxacin and ciprofloxacin. Such a high resistance profile could possibly be a consequence of incomplete dosage and unchecked prescription. Based on our study, alternate therapy has to be followed as quinolones are no longer advisable for treatment of UTIs. Global surveillance studies have ascertained a rise in the incidence of community acquired UTIs and intraabdominal infections owing to fluoroquinolone resistance in Enterobacteriaceae. Although antimicrobial susceptibility patterns may differ geographically, data collected from regions of Asia-Pacific and India show that quinolone resistant uropathogens are increasing at an alarming rate [16].

Many reports confirm mutations in codons 83 and 87 of gyrase A to be responsible for resistance to quinolones [27, 28]. In the present study, sensitive strains contained Ser83Leu, which could be evidence that it is not responsible for resistance alone. It is only in the presence of a second mutation that resistance developed. Double mutants show a 10-fold increase in MIC levels than sensitive strains. A larger sample size to confirm the presence of *gyrA* mutation (Ser83Leu) would have benefitted the study.

Previous findings suggest GyrA residues 83 and 87 are crucial for interaction with quinolones [24, 29]. We observed the effect of GyrA mutations on quinolone binding through docking studies. Displacement of the binding site resulted in a lower binding energy as compared to the normal protein complex. This could be because of less specific interaction with the mutated protein which could also explain its correlation with the high resistance pattern among *E*.*coli* isolates. A detailed molecular study of structure-activity relationship of quinolones is a requisite to counteract the problem of resistance.

To overcome the problem of quinolone resistance, molecular docking trials have been performed with novel ligands that target GyrA outside the region of QRDR [30]. Diones like quinazolinediones have also been investigated through docking studies for their ability to bypass the GyrA-mediator resistant mutations [31]. However with novel drugs yet to be approved for replacement of fluoroquinolones, molecular docking could be used to provide elaborate insight into the interaction of second and third generation quinolones with DNA gyrase; and thus enhance the accuracy of drug prescription.

## Supporting information

S1 Fig Phylogenetic tree of *gyrA* QRDR of *E*. *coli* strains. (TIF)
