# Supplementary figures and images for: Analytical profiling of mutations in quinolone resistance determining region of gyrA gene among UPEC
Source: PLoS One. 2018 Jan 4;13(1):e0190729. doi: 10.1371/journal.pone.0190729 (PMC5754135; doi:10.1371/journal.pone.0190729)

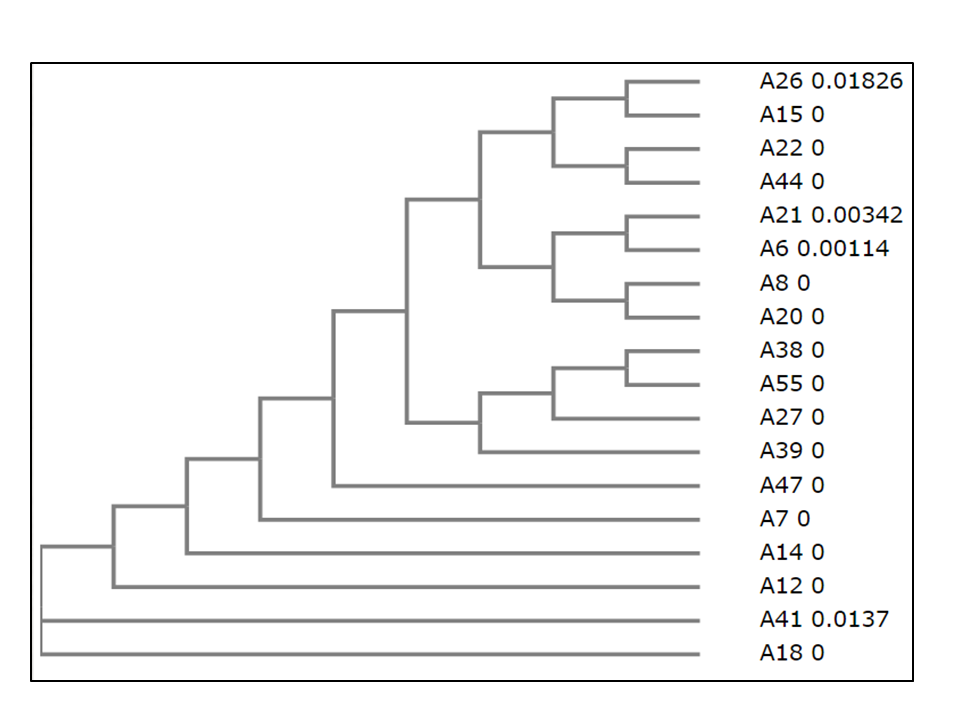

Supplement: S1 Fig — (TIF) [file pone.0190729.s001.tif]
